# An Innovative and Alternative Technique in Total Colonic Hirschsprung Disease (TCHD) Treatment, Two Case Reports

**DOI:** 10.1002/ccr3.73166

**Published:** 2026-07-17

**Authors:** Ali Khaksour, Jamshid Yousefi, Seyed Mohammad Ali Raisolsadat

**Affiliations:** ^1^ Clinical Research Development Unit of Akbar Hospital, Faculty of Medicine Mashhad University of Medical Sciences Mashhad Iran; ^2^ Department of Pediatrics MMS, Islamic Azad University Mashhad Iran; ^3^ Department of General Surgery MMC, Islamic Azad University Mashhad Iran

**Keywords:** case report, Hirschsprung disease, surgical technique, total colonic Hirschsprung disease

## Abstract

With reported occurrences ranging from 1% to 20%, total colonic Hirschsprung disease (TCHD) accounts for about 8% of Hirschsprung disease cases. The selection and sequencing of the surgical approach, access to parenteral nutrition, and collaborative care between surgeons, gastroenterologists, and pathologists are all critical factors in achieving optimal long‐term outcomes. Currently, there is no consensus on the best way to treat TCHD patients. Despite the accompanying morbidity and mortality, standardization of care is required.

## Key Clinical Message

This method involves mobilizing a sufficient length of ganglionated terminal ileum for an ileoanal anastomosis in soave procedure and construction hybrid pouch with sigmoid after resection the rest of Aganglionic colon. The distal ileum (at least 10 cm) is then passed through the rectal sleeve for the anastomosis. Key advantages of this approach are discussed in the paper context.

## 1 Introduction

With reported occurrences ranging from 1% to 20%, total colonic Hirschsprung disease (TCHD) accounts for about 8% of Hirschsprung disease (HD) cases [1, 2]. It affects boys more often than females, with a male‐to‐female ratio of 1.5:1 to 2:1 [3], and its incidence is approximately 1 in 100–200K births [4]. However, there is little and inconsistent research on the surgical management of children with TCHD, both before and after surgery. The need for a systematic approach to care for this unique patient group is underscored by high complication rates. Twenty years ago, the mortality rate for TCHD was over 60% [5, 6], and in some studies, it was as high as 100% [5]. Half of the deaths occurred before surgery [7]. The mortality rate has been reduced to approximately 11% due to improved perioperative care. These fatalities are attributed to factors that are not directly related to the disease, such as dehydration, line sepsis, and poor nutrition [8, 9].

The selection and sequencing of the surgical approach, access to parenteral nutrition, and collaborative care between surgeons, gastroenterologists, and pathologists are all critical factors in achieving optimal long‐term outcomes. Currently, there is no consensus on the best way to treat TCHD patients. Despite the accompanying morbidity and mortality, standardization of care is required. The logical analysis of specialists supports the suggestions made.

## 2 Case Presentation

### 2.1 Case1

The parents of a six‐month‐old boy who had an emergency colostomy during the neonatal period brought him to our hospital for colostomy closure after he was referred to from a rural health center. The parents' history states that on day ten of birth, surgery was done to externalize the intestine (colostomy) in response to an apparent intestinal perforation. There was no family history of illness or afflicted siblings, and the baby was born full‐term. Weight was within age‐appropriate ranges, vital signs were within normal ranges, and the colostomy was operating as intended. In the transverse colon, close to the midline, was the stoma. A cardiologist's assessment of the heart revealed no anomalies, and no pathological signs were detected in any other system.

The medical records, operational notes, and prior investigations from the hospital where the emergency surgery was performed should be obtained by the parents, as the etiology of the neonatal colostomy remains uncertain. When the parents returned ten days later, they explained that the prior surgeon had not been available and that ongoing hospital renovations had prevented them from obtaining the records. Given the potential for the neonatal colostomy to have been performed for an underlying condition, such as necrotizing enterocolitis or Hirschsprung's disease, a surgical anorectal biopsy was advised to rule out Hirschsprung's disease because a suction biopsy was not available.

Following thorough preoperative planning, a rectal biopsy was carried out and submitted for pathological analysis. Hirschsprung's illness was ruled out when ganglion cells were discovered using permanent slice examination. To locate any underlying pathology, colostomy closure with exploratory laparotomy was planned, considering this discovery. The patient had surgery using a left upper abdominal incision; the splenic flexure was mobilized, the colostomy site was resected, and a Colo‐colic anastomosis was carried out. There were no other intra‐abdominal anomalies found—colostomy site excision sent for pathologic examination. By the third postoperative day, the patient had passed stool, was able to tolerate feeding, and was discharged. After one week, the pathology result was recorded as suspected aganglionosis; however, the patient's family was not reachable due to nomadic migration.

The baby arrived at the clinic fifteen days after surgery in terrible overall health, with fever, diarrhea, and abdominal distension. He also looked extremely toxic and dehydrated. Treatment started when he was brought up urgently with a presumed diagnosis of Hirschsprung Associated Enterocolitis (HAEC). Suspicions of abscess formation or intestinal necrosis led to the decision to do a diagnostic laparotomy after 24 h without improvement and ongoing acute toxicity. The right colon had an ischemic appearance and necrosis, which resembled typhlitis, according to intraoperative observations. A right hemicolectomy with ileostomy was performed; the previous anastomosis was taken down, and a Hartmann's procedure was completed. Several seromuscular samples were obtained from the rectum and sigmoid colon due to their aberrant thickness.

The patient's condition improved after ileostomy and continued medical care, and intestinal transit through the ileostomy was established in less than a week. After enduring feeding, the patient was released. The initial underlying diagnosis of Hirschsprung's illness was confirmed by pathology from the sigmoid and rectal seromuscular biopsies, which showed no ganglion cells. There was no explanation for why the first anorectal biopsy revealed ganglion cells. Although, finally, since we send pathology specimens to another hospital for evaluation by a pediatric pathologist to ensure diagnostic accuracy, subsequent follow‐up and investigation revealed that the error was due to incorrect specimen labeling by the nursing staff.

The next course of treatment involved creating an ileal pouch with an ileoanal anastomosis, as the right colon had been removed due to necrosis, and the remaining left colon lacked ganglion cells. With the ileostomy in place, the patient was released, and two months later, definitive surgery was planned with parents' consent.

**FIGURE 1:**
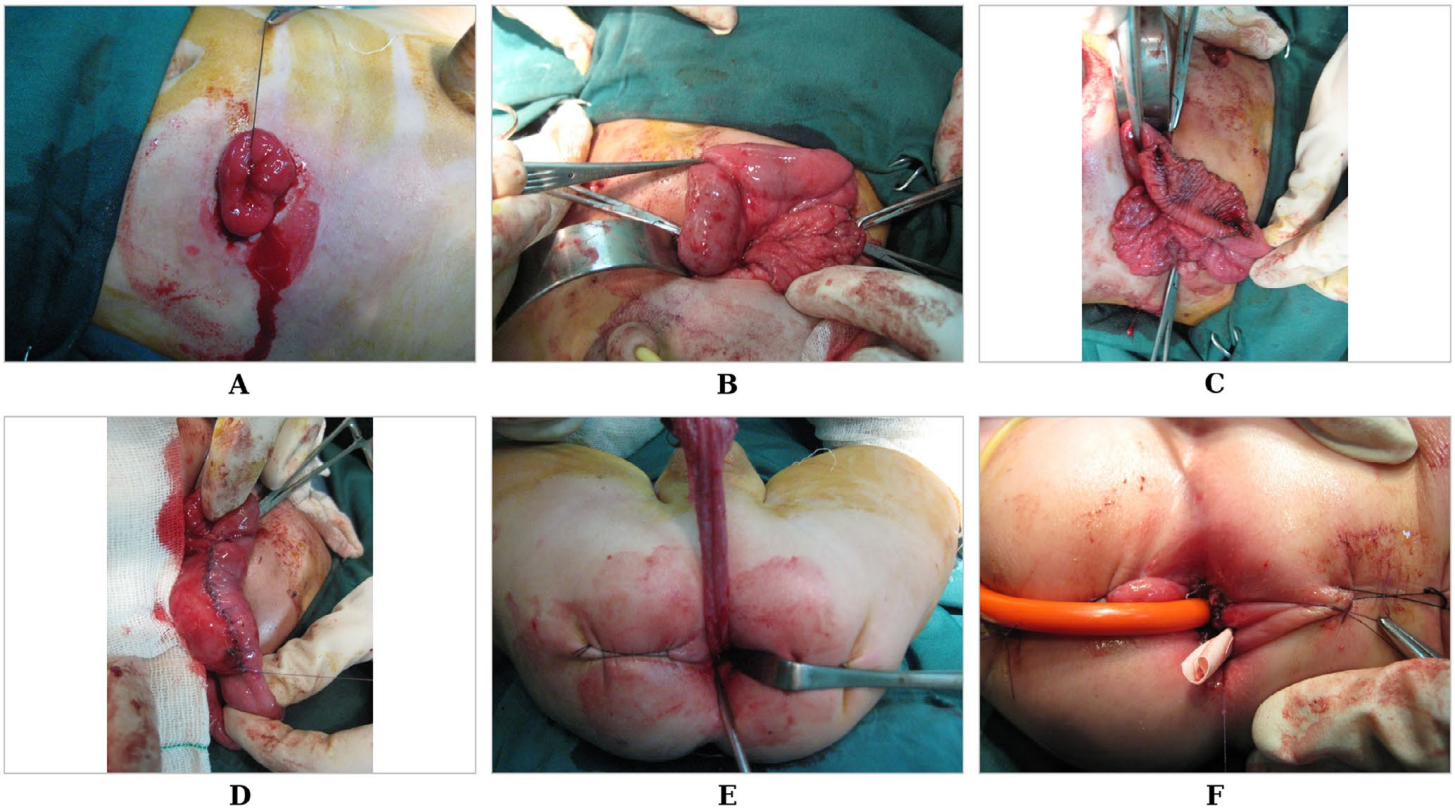
Ileostomy site (A), ilium and sigmoid preparation (B), ilium and sigmoid antimesenteric incision (C), ilio‐sigmoid pouch reconstruction (D), rectal mucosectomy (E), ilio‐anal anastomosis (F).

**FIGURE 2:**
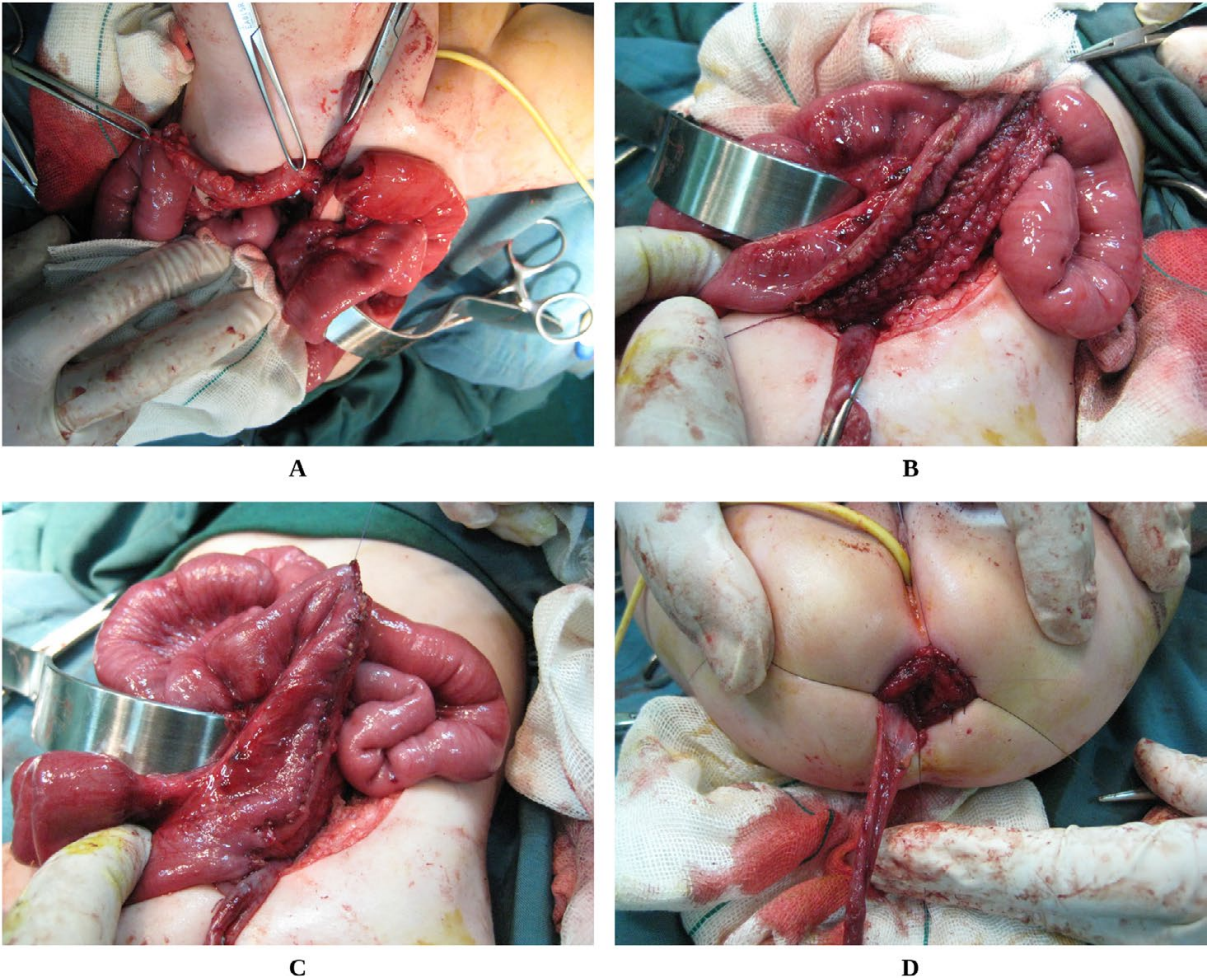
Ilium and sigmoid preparation (A), Ilium and sigmoid incision and pouch reconstruction (B), Reconstructed pouch (C), Transrectal Ilio‐anal anastomosis (D).

The patient was brought to the operating room two months later, under ideal circumstances, in a baby with normal range weight and normal preoperative lab tests, and good ileal defecation consistency. After thorough parental counseling regarding the potential risks of ileal pouch surgery and long‐term care. A hybrid strategy was developed, considering the age, clinical state, and long‐term issues associated with conventional ileal pouch–anal anastomosis. Rather than building the pouch entirely of ileum, a combination pouch was made from both diseased sigmoid colon and ileum (Martin like technique). The distal ileum (about 10 cm long) was anastomosed into the anus by using a Soave sleeve dissection, which conceptually resembled the Soave operation distally (Figures 1 and 2).

All these staged surgeries have been done with the patients' parents' written consent under a legal order.

The postoperative period went smoothly. By the third postoperative day, bowel transit had returned, and precise fluid feeding had been started. On the seventh day, the patient was released. The patient followed up monthly until 3 years and toilet training age and fecal continency. The patient didn't find diaper rash required long term treatment (G1, 2 in Clavien‐Madadi Classification) [10]. No diarrhea or enterocolitis with good continency of solidity and gas. These might be due to nutritional culture to use more fiber and fruit in the region. Seven years after surgery, the youngster is now asymptomatic, has no surgery‐related problems, and is enrolled in school.

We suggest this hybrid approach as a possible alternative for the Kimura or Martin procedures and conventional ileal J‐pouch reconstruction in the treatment of Total Colonic Hirschsprung's Disease, considering this positive result.

### 2.2 Case 2

We present a case involving twin neonates, one male and one female, who were both suspected of having Hirschsprung's disease due to delayed meconium passage within the first 48 h of life and progressive abdominal distension. A contrast enema revealed delayed evacuation of contrast material beyond 48 h. In addition, repeated normal saline enemas and colonic washouts were unsuccessful in producing adequate stool output, and abdominal distension worsened. Consequently, colostomy was planned for both neonates during the first week of life.

Following complete preoperative assessment, including cardiac, hematologic, and electrolyte evaluations, the infants were taken to the operating room. Intraoperative full‐thickness biopsies were obtained, frozen sections were examined, and additional samples were sent for permanent histopathology. In the male neonate, aganglionosis was found to be limited to the sigmoid colon. A proximal sigmoid colostomy was created in a normally innervated segment. In the female neonate, multiple biopsies with frozen sections and then in permanent pathology demonstrated the absence of ganglion cells extending to the ileocecal valve; therefore, an ileostomy was performed during the neonatal period.

Postoperatively, both stomas functioned well, and the patients were discharged in stable condition. Definitive surgery was scheduled for after the age of six months. The male infant underwent a Soave pull‐through procedure. For the female infant, given the extensive aganglionosis and the challenges associated with standard ileoanal anastomosis and J‐pouch formation, a hybrid approach was planned. With informed parental consent and under appropriate antibiotic coverage, with normal weight and 4‐time daily ileostomy defecation, the procedure involved creating a hybrid pouch using an aganglionic sigmoid colon and normal ileum. After preparing the ileum and performing a rectal sleeve dissection without pelvic manipulation, the hybrid pouch was constructed approximately 10 cm proximally to the distal ileum, and an ileoanal anastomosis via the rectal sleeve was performed in a Soave‐like fashion (Figure 2).

The surgery has been done with the patient's parents' written consent under legal order.

The postoperative course of the hybrid procedure was excellent. To prevent anal anastomotic stricture, daily dilatation with a dilator was performed for two weeks. The female infant maintained good bowel function, gained weight appropriately, and experienced no gastrointestinal complications and monthly follow‐up until the age of 2 years. Unfortunately, at 25 months, she was admitted to the cardiology service with a diagnosis of endocarditis. At this time the infant did not have any gastrointestinal symptoms of eventual enterocolitis. However, she died due to possible sepsis, as her cardiologist told, despite negative blood cultures, based on echocardiographic findings that were suggestive of the condition.

## 3 Technique

In this method, schematically illustrated in the figures, an adequate length of terminal ileum—confirmed to be ganglionated—is mobilized for use in the intended technique. The colon is resected up to the sigmoid segment, proximal to the anatomical point of Sudeck. Distally, starting from the anterior aspect of the rectosigmoid junction, rectal mucosectomy is performed (creating the Soave sleeve technique). After determining the required length of ileum (at least 10 cm) for the ileoanal anastomosis from the distal end, the ileum is incised along its antimesenteric border, and an appropriate length of aganglionic sigmoid colon is incised (about 10 cm). A hybrid pouch is then constructed from the ileum and sigmoid colon. Following pouch creation, the distal ileum is passed through the rectal sleeve, and an ileoanal anastomosis is performed.

**FIGURE 3:**
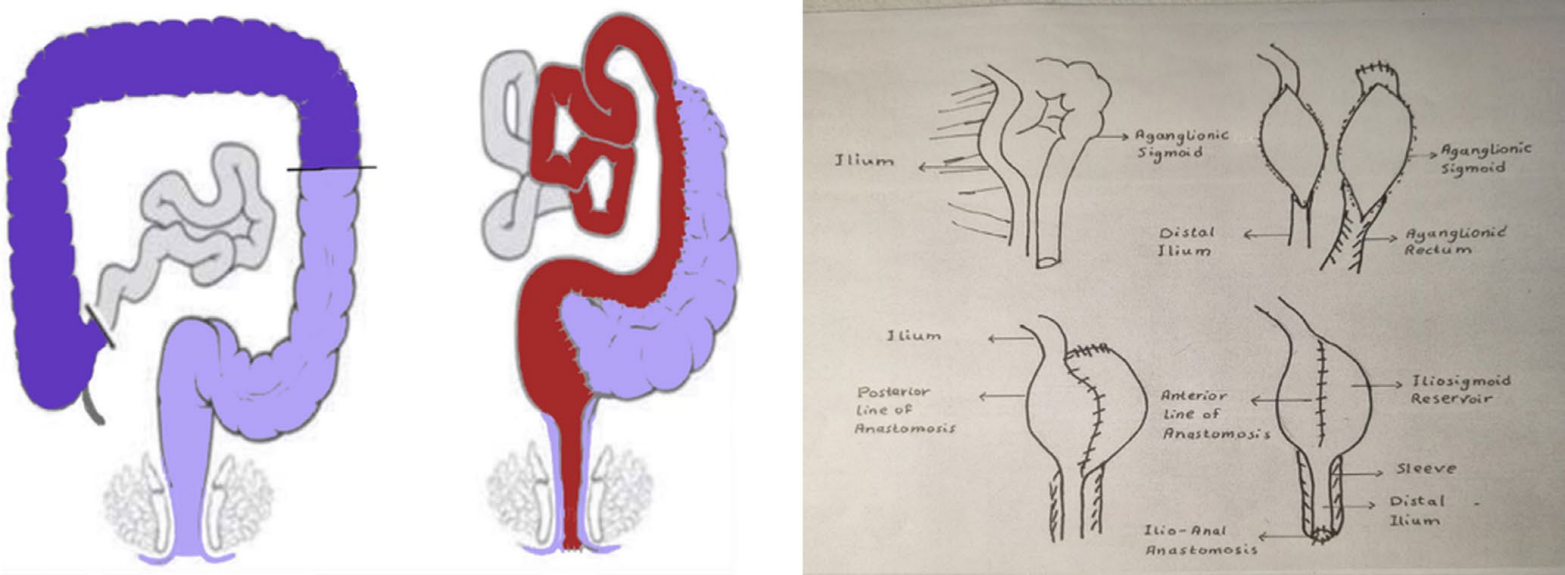
Drawing the schematic procedure map.

The advantages of this approach include the possibility of completion in a single stage, the absence of complications typically associated with an ileal J‐pouch, no manipulation in the pelvis and neural plexus, no manipulation of the vascular arcade, and a better J pouch reservoir for water and electrolytes absorption (Figure 3).

## 4 Discussion and Conclusion

Although the definition of long‐segment Hirschsprung disease (HSCR) varies across the literature, the *American Pediatric Surgical Association Outcomes and Evidence‐Based Practice Committee* defines long‐segment disease as aganglionosis extending proximally from the sigmoid–descending colon junction toward the cecum, with ganglion cells present in at least part of the colon. In contrast, total colonic aganglionosis (TCA) is defined as aganglionosis involving the entire colon with less than 5 cm of distal small bowel involvement, whereas small‐bowel HSCR involves more than 5 cm of aganglionic small intestine [1].

TCA accounts for approximately 5%–15% of all HSCR cases and occurs in approximately 1 in 50,000 live births [11]. Unlike classic HSCR, which demonstrates a marked male predominance, TCA occurs with nearly equal frequency in both sexes and is more commonly associated with a positive family history [12].

Compared with short‐segment disease, TCA is associated with substantially greater morbidity and mortality. Patients frequently develop complications both before and after definitive surgery, including Hirschsprung‐associated enterocolitis (HAEC), severe diarrhea, electrolyte disturbances, fecal incontinence, growth failure, recurrent infections, and prolonged nutritional impairment. Consequently, TCA management remains surgically and medically challenging and requires long‐term multidisciplinary follow‐up [13].

Initial treatment typically consists of decompression with a diverting ileostomy using the most distal ganglionated bowel. Intraoperative mapping biopsies are essential at the time of the initial procedure to accurately define the proximal extent of aganglionosis and determine the optimal site for stoma creation, thereby minimizing the risk of future stoma revision [14, 15].

Although criteria for timing of definitive reconstruction are relatively well established—including adequate weight gain, ostomy output < 30 mL/kg/day, apple‐sauce stool consistency, and urine sodium > 20 mmol/L [16]—there remains no universally accepted operative time and approach for TCA.

Given the rarity of the disease, with an estimated incidence of 1 in 100,000–200,000 live births, most pediatric surgeons encounter only a small number of cases during their careers [17, 18]. As a result, operative experience and technical preference vary considerably among institutions.

In a clinical consensus statement within ERNICA, *the European Reference Network for rare Congenital digestive diseases*, due to a lack of evidence, no unequivocal expert opinion was reached regarding whether one specific pull‐through procedure is preferable over others. The panel of ERNICA concurred that the pull‐through technique should be chosen based on the experience of the operating surgeon [19, 20].

**FIGURE 4:**
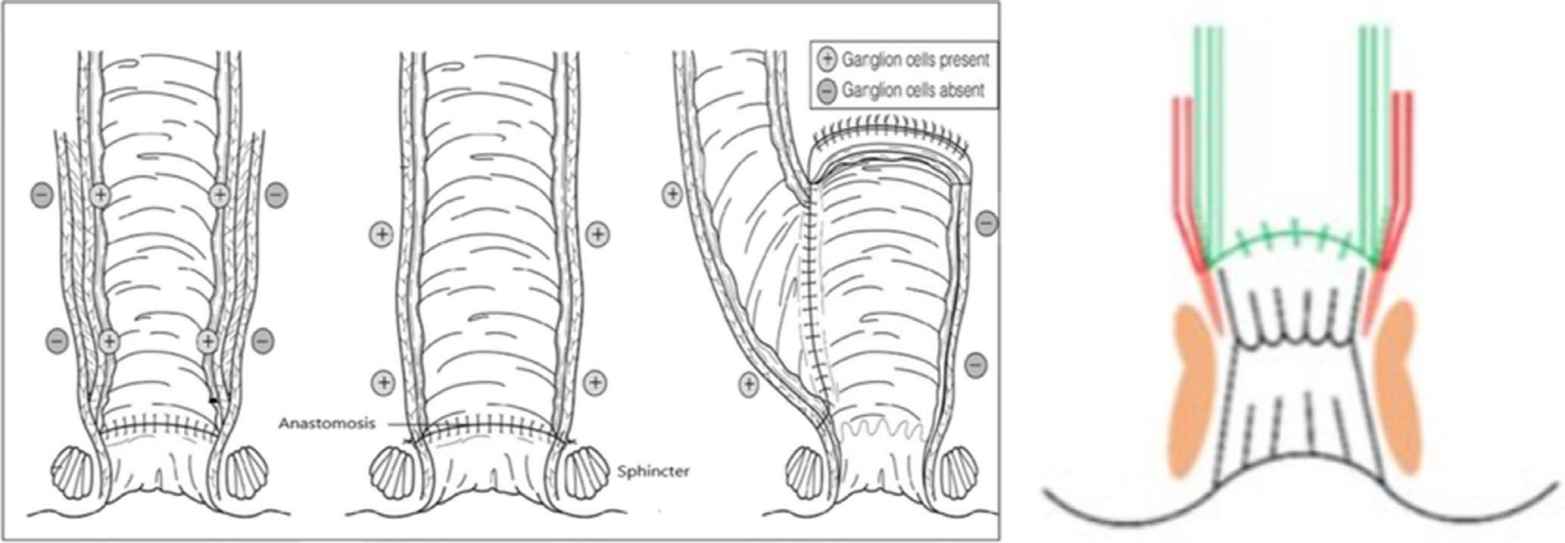
From left to right: an endorectal dissection (Soave), full‐thickness rectosigmoid dissection (Swenson), a retrorectal pouch procedure (Duhamel) [21] and Vancy Soave technique [22].

Several operative techniques have historically been described for the management of TCA, including the Soave, Swenson, Duhamel, and Yancey–Soave procedures. (Figure 4).

Subsequent modifications were developed to reduce complications such as anastomotic leakage, enterocolitis, poor continence, and unfavorable long‐term functional outcomes [23, 24].

**FIGURE 5:**
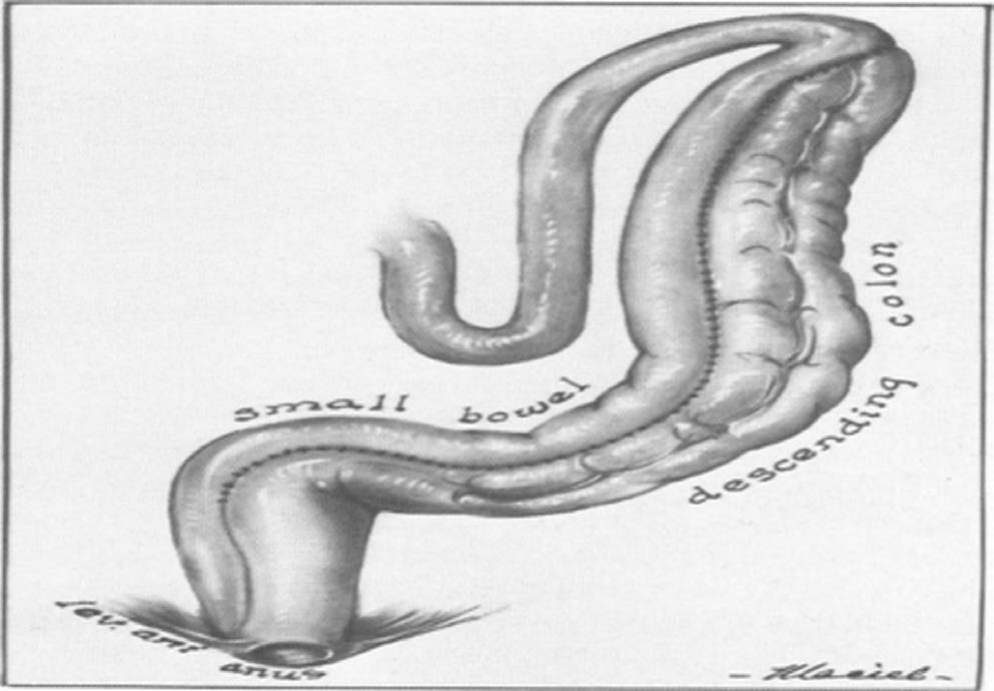
Martin's ileocolostomy (descending colon)—original work [26].

**FIGURE 6:**
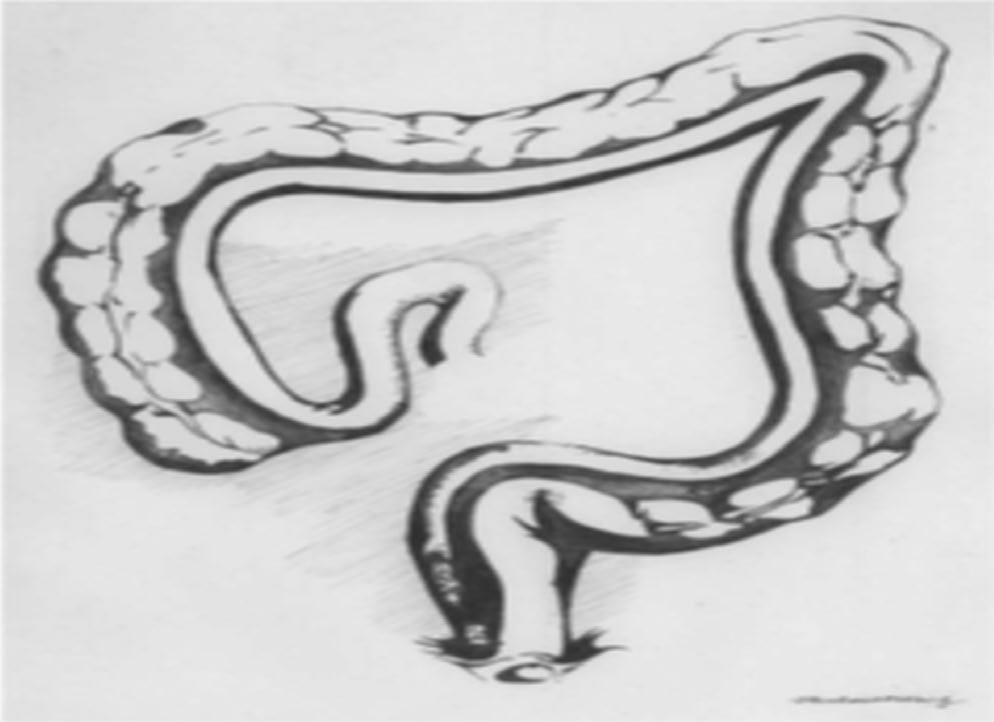
Modified martins ileocolostomy (total colon) [9].

Martin et al. described a modification utilizing the absorptive capacity of retained aganglionic colon through creation of a side‐to‐side ileocolostomy with the descending and sigmoid colon [25] (Figure 5). This technique was later modified to incorporate the entire colon due to complications like diarrhea [9, 26] (Figures 5 and 6).

Our approach shares certain conceptual similarities with the Martin procedure in that the left colon and sigmoid colon are preserved for reservoir formation. However, unlike the Martin technique, extensive pelvic dissection was avoided in our procedure.

**FIGURE 7:**
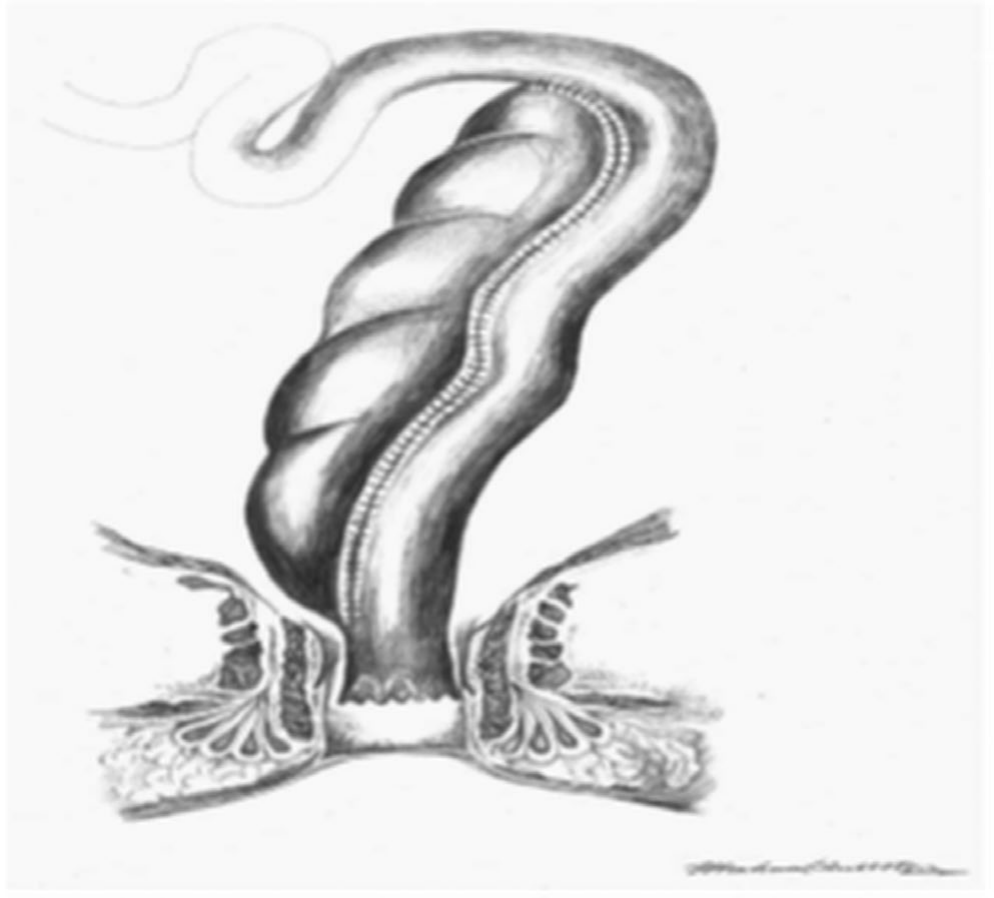
Ascending colon patch—original work (Boly).

Boley [26] subsequently introduced the ascending colon patch procedure in an effort to reduce the severe diarrhea frequently observed following Martin‐type operations (Figure 7). In this technique, a segment of ascending colon is anastomosed to ganglionated ileum while maintaining vascular supply through the ileocolic pedicle. However, the procedure differs substantially from our approach and may carry a risk of ischemic complications during staged reconstruction.

**FIGURE 8:**
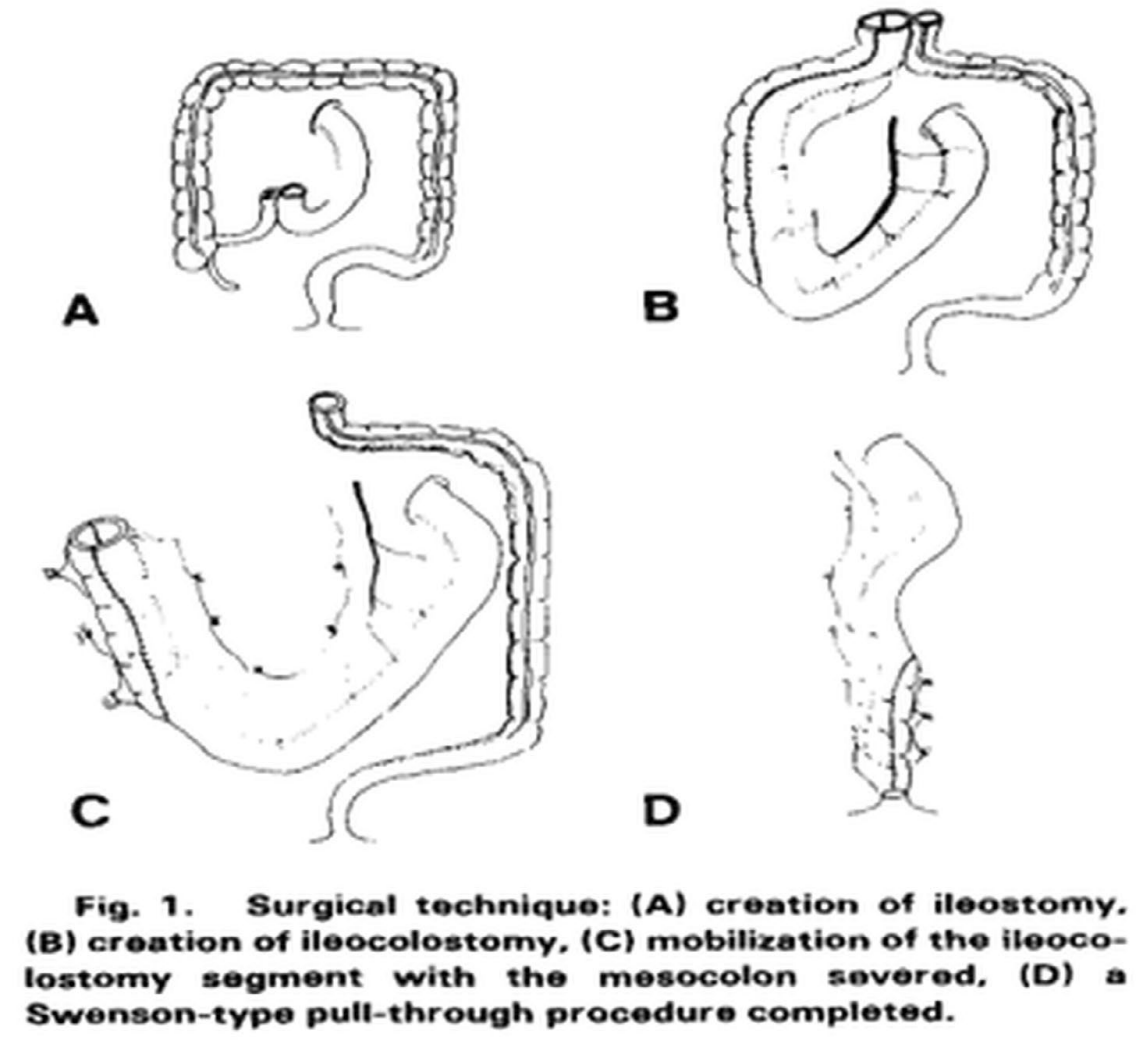
Kimura ileocolostomy (ascending colon) [27].

Kimura [27] later described a two‐stage reconstruction consisting of right ileocolostomy combined with a Swenson pull‐through (Figure 8). In contrast to our technique, Kimura's procedure does not incorporate a Soave‐type rectal cuff and needs pelvic manipulation.

**FIGURE 9:**
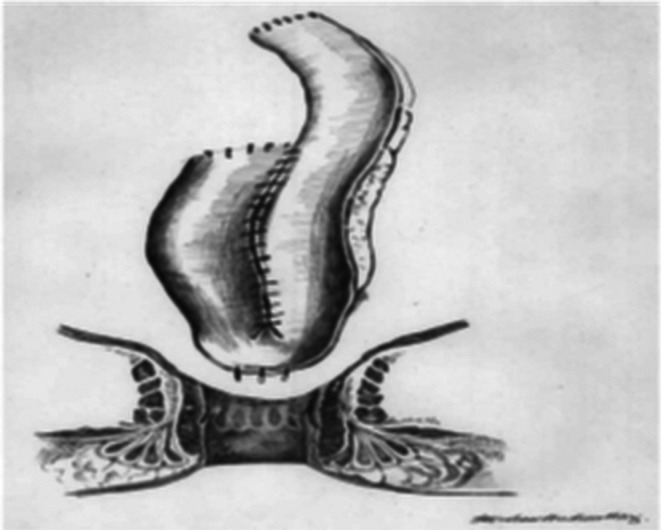
Proctocolectomy and J‐pouch ileo‐anal anastomosis [28].

Ileal J‐pouch anal anastomosis (Figure 9), widely utilized in ulcerative colitis surgery, has also been applied in selected patients with TCA [28].

A long‐stapled J‐pouch is formed from the distal ileum with a single layer handsewn ileoanal anastomosis (Figure 9). Although this technique may improve reservoir capacity, complications including pouchitis, high stool frequency, severe perianal excoriation, and chronic diarrhea remain significant concerns and long‐term care [29, 30].

Experimental work by Heath et al. demonstrated prolonged intestinal transit time and improved sodium and water absorption in animal models incorporating aganglionic colon patches compared with isolated ileoanal anastomosis [31]. These findings suggest that preservation of aganglionic colonic segments may confer physiologic advantages in selected patients.

**FIGURE 10:**
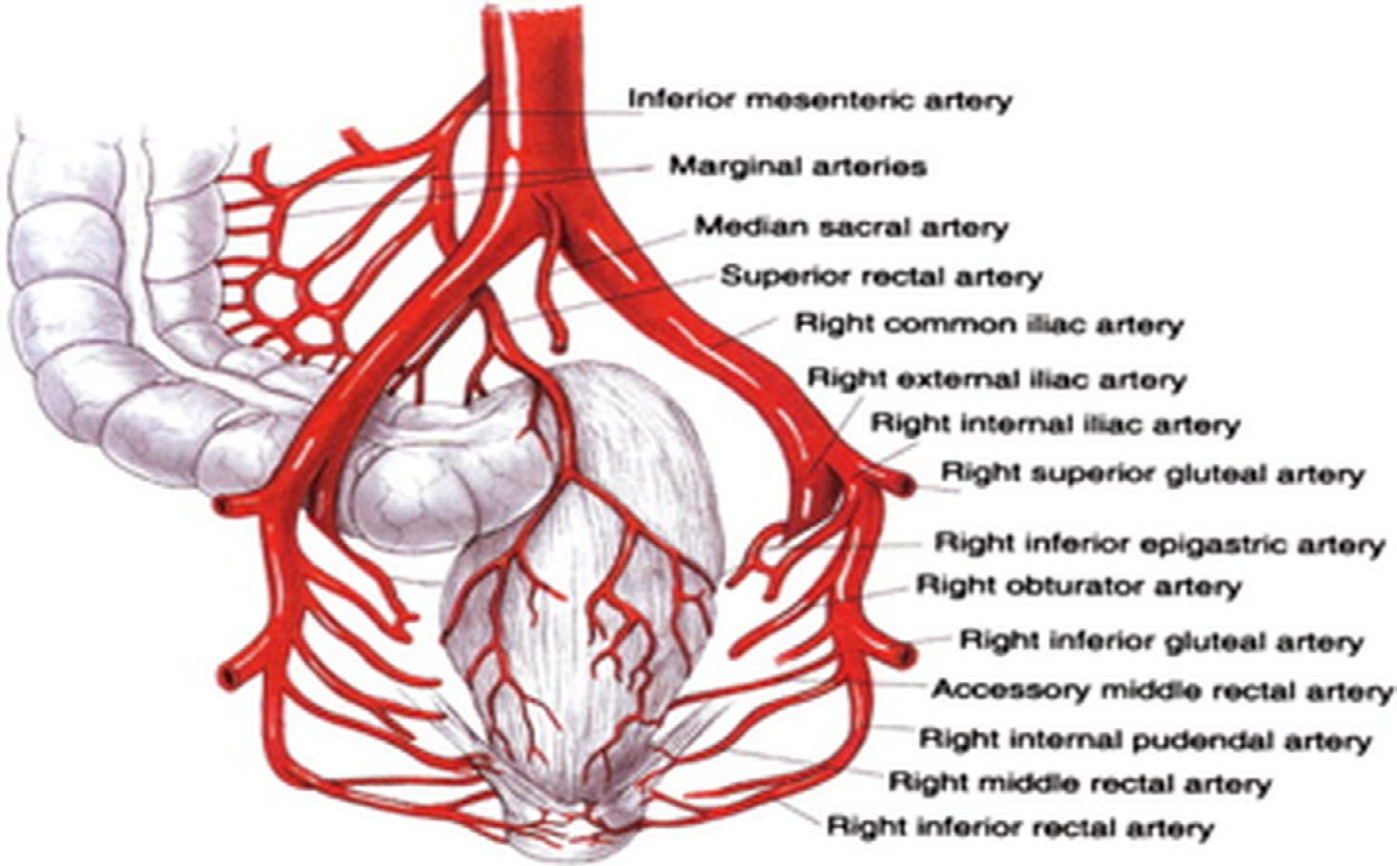
Vascular anatomy of the inferior mesentery shows Sudeck's critical point and the superior rectal artery, which supplies the sigmoid colon and rectum and is preserved in our technique.

An additional and potentially important aspect of our technique is preservation of the vascular anatomy of the rectosigmoid and anorectal region, an issue that has received relatively little attention in previous reports. Blood supply to this region is derived primarily from the superior rectal artery, the terminal branch of the inferior mesenteric artery through the sigmoid arterial arcade [32] (Especially Sudeck and Hartmann critical points), although the considerable anatomic variability of this vascular network, minimizing dissection in this region may reduce the risk of ischemic complications and preserve anorectal function. Avoidance of extensive pelvic dissection may also reduce the likelihood of pelvic nerve injury (Figure 10) [33].

Based on the aforementioned considerations, we propose this technique as an alternative surgical approach for the management of total colonic Hirschsprung disease (TCHD).

First, to the best of our knowledge, the technique described in its present form has not previously been reported in the literature. In contrast to the Martin, Boley, and Kimura procedures [9], our approach preserves at least 10 cm of distal ganglionic ileum, whereas the distal reconstructed segment in those techniques consists of a relatively large aganglionic colonic patch and a smaller ganglionic ileal segment.

One of the recognized limitations of the Martin procedure has been persistent postoperative diarrhea [34]. The rationale for using the right colon as a patch in that technique was to enhance water and electrolyte absorption [26]. In our method, however, the vascular supply of the colonic patch is preserved without extensive vascular manipulation, potentially reducing the risk of ischemia and patch necrosis compared with techniques utilizing right colonic patches [32, 35].

An additional advantage of this approach is the avoidance of prolonged proximal fecal diversion, which may impose considerable physical, psychological, and financial burdens on families [30]. Furthermore, in the event of severe or unmanageable complications, conversion to a terminal ileoanal anastomosis—currently regarded as the definitive salvage procedure—can be performed relatively easily by excising the colonic patch without requiring further pelvic or sphincter dissection.

Although distal ileostomy might remain an accepted management strategy for TCHD, it frequently necessitates prolonged postoperative care, including regular trans‐anal irrigations and stoma‐related management, which may be particularly challenging in low‐resource settings [29, 30]. Despite their technical complexity, patch procedures continue to represent viable alternatives within the surgical armamentarium of pediatric colorectal surgeons.

We therefore present this technique as an alternative reconstructive option that warrants further clinical experience and long‐term evaluation. Notably, several comparative studies from Europe and North America have failed to demonstrate clear superiority of any single operative technique for TCHD, with procedural selection remaining largely dependent on surgeon preference and institutional experience [9, 19].

Nevertheless, additional experience, larger patient cohorts, and longer follow‐up are required before this technique can be considered a standard surgical option in the management of total colonic aganglionosis.

This case report was prepared and reported with the permission of the university's vice chancellor for research and the ethics committee, and the personal informed consent of the patients' parents.

## Author Contributions

**Ali Khaksour:** data curation, investigation, writing – original draft, writing – review and editing. **Jamshid Yousefi:** data curation, investigation, writing – original draft, writing – review and editing. **Seyed Mohammad Ali Raisolsadat:** conceptualization, data curation, investigation, writing – original draft, writing – review and editing.

## Funding

The authors have nothing to report.

## Ethics Statement

Data is available from the author(s) with the permission of informed consent of both patient's parents separately.

## Consent

Informed consents was received from all Patients parents.

## Conflicts of Interest

The authors declare no conflicts of interest.

## Data Availability

The data that support the findings of this study are fully open available in doi of manuscript (https://10.1002/ccr3.73166).
